# Readiness and Acceptance of eHealth Services for Diabetes Care in the General Population: Cross-sectional Study

**DOI:** 10.2196/26881

**Published:** 2021-09-02

**Authors:** PV AshaRani, Lau Jue Hua, Kumarasan Roystonn, Fiona Devi Siva Kumar, Wang Peizhi, Soo Ying Jie, Saleha Shafie, Sherilyn Chang, Anitha Jeyagurunathan, Chua Boon Yiang, Edimansyah Abdin, Janhavi Ajit Vaingankar, Chee Fang Sum, Eng Sing Lee, Siow Ann Chong, Mythily Subramaniam

**Affiliations:** 1 Research Division Institute of Mental Health Singapore Singapore; 2 Admiralty Medical Centre Khoo Teck Puat Hospital Singapore Singapore; 3 Clinical Research Unit National Healthcare Group Polyclinics Singapore Singapore; 4 Saw Swee Hock School of Public Health National University of Singapore Singapore Singapore

**Keywords:** eHealth, diabetes, general population, acceptance, readiness

## Abstract

**Background:**

Diabetes management is a growing health care challenge worldwide. eHealth can revolutionize diabetes care, the success of which depends on end user acceptance.

**Objective:**

This study aims to understand the readiness and acceptance of eHealth services for diabetes care among the general population, perceived advantages and disadvantages of eHealth, and factors associated with eHealth readiness and acceptance in a multiethnic Asian country.

**Methods:**

In this cross-sectional epidemiological study, participants (N=2895) were selected through disproportionate stratified random sampling from a population registry. Citizens or permanent residents of Singapore aged >18 years were recruited. The data were captured through computer-assisted personal interviews. An eHealth questionnaire was administered in one of four local languages (English, Chinese, Malay, or Tamil), as preferred by the participant. Bivariate chi-square analyses were performed to compare the sociodemographic characteristics and perception of advantages and disadvantages of eHealth services between the diabetes and nondiabetes groups. Multivariable logistic regression models were used to determine factors associated with eHealth readiness and acceptance. All analyses were weighted using survey weights to account for the complex survey design.

**Results:**

The sample comprised participants with (n=436) and without (n=2459) diabetes. eHealth readiness was low, with 47.3% of the overall sample and 75.7% of the diabetes group endorsing that they were not ready for eHealth (*P*<.001). The most acceptable eHealth service overall was *booking appointments* (67.4%). There was a significantly higher preference in the diabetes group for face-to-face sessions for *consultation with the clinician* (nondiabetes: 83.5% vs diabetes: 92.6%; *P*<.001), *receiving prescriptions* (61.9% vs 79.3%; *P*<.001), *referrals to other doctors* (51.4% vs 72.2%; *P*<.001), and *receiving health information* (34% vs 63.4%; *P*<.001). The majority of both groups felt that eHealth requires users to be computer literate (90.5% vs 94.3%), does not build clinician-patient rapport compared with face-to-face sessions (77.5% vs 81%), and might not be credible (56.8% vs 64.2%; *P*=.03). Age (≥35 years), ethnicity (Indian), and lower education status had lower odds of eHealth readiness. Age (≥35 years), ethnicity (Indian), lower education status (primary school), BMI (being underweight), and marital status (being single) were associated with a lower likelihood of eHealth acceptance. Among only those with diabetes, a longer duration of diabetes (4-18 years), higher education (degree or above), and younger age (23-49 years) were associated with eHealth readiness, whereas younger age and income (SGD 2000-3999 [US $1481-$2961]) were associated with acceptance.

**Conclusions:**

Overall, an unfavorable attitude toward eHealth was observed, with a significantly higher number of participants with diabetes reporting their unwillingness to use these services for their diabetes care. Sociodemographic factors associated with acceptance and readiness identified a group of people who were unlikely to accept the technology and thus need to be targeted for eHealth literacy programs to avoid health care disparity.

**International Registered Report Identifier (IRRID):**

RR2-10.1136/bmjopen-2020-037125

## Introduction

### Background

Diabetes has significant social and economic consequences globally [1]. The prevalence of diabetes increased from 422 million in 2016 [1] to 463 million in 2019 [2], despite innovative disease prevention strategies [1]. This number is estimated to increase by 51% (to 700 million) by 2045 [2], which will lead to higher consumption of health care services [3]. Health care systems are not equipped to manage such a surge in the number of cases [4] and are expected to face additional challenges in the coming years, as a shortage of trained staff is expected because of falling birth rates [5]. The geographical isolation of people living in remote areas is another challenge for health care delivery [6]. This situation calls for a change in the way patients are managed without compromising the quality of health care services.

eHealth is defined by the World Health Organization as a “cost-effective and secure use of information technology in health-related fields: health care services, health surveillance, health literature, and health education, knowledge and research” [7]. Patients with diabetes require regular monitoring of their blood glucose levels, diet, physical activities, and medications to achieve glycemic control [8,9], which will reduce their risk of mortality and cardiovascular complications [10]. eHealth offers diverse treatments and management options in a cost-effective manner to facilitate these requirements [11] and thus allows both clinicians and patients to manage the disease efficiently in a collaborative manner. A meta-analytic review of 18 randomized controlled trials showed a significant reduction in glycated hemoglobin and better glycemic control in participants using eHealth compared with those monitored through routine methods [12]. Pereira et al [13] reviewed the literature on the delivery of health education through eHealth and concluded that the technology delivered better outcomes in terms of efficient management of diabetes. eHealth platforms can also be used for promoting a healthy lifestyle in patients with diabetes by facilitating exercise lessons through video games or other virtual environments [14,15]. Apart from the remote management of diseases, eHealth also offers services such as electronic prescriptions, referrals, teleconsulting, and health education. Thus, eHealth empowers patients to manage their disease and make decisions on their health care remotely, without the need to travel to hospitals.

An individual’s decision to use or accept a technology is based on their perception of usefulness and ease of use, which shapes their attitude toward eHealth and intention to use the technology (Technology Acceptance Model). A positive attitude is an antecedent to intention or readiness to use the service. Thus, by modifying the perception of usefulness and ease of use, acceptance can be improved [16,17]. Hossain et al [17] reported that a positive attitude toward eHealth, perceived effectiveness, and access to cell phones were associated with eHealth acceptance in developing countries. The authors also showed that a positive attitude toward technology increased the odds of acceptance by 4.5-fold and reported that sociodemographic factors such as age, gender, and education have a significant influence on this choice. A study of patients with chronic respiratory disease reported higher acceptance of eHealth services for booking appointments, accessing laboratory results, educational purposes, and receiving e-prescriptions, than acceptance of eHealth for treatment-related services such as contact with the health care team and referrals to other clinicians [18]. The likelihood of acceptance was dependent on the duration of the disease, age, and education. A subsequent large-scale study from the author further confirmed that education and perceived usefulness determined eHealth acceptance [19]. Thus, the role of sociodemographic determinants, perception of usefulness, and ease of use in eHealth acceptance is irrefutable.

Although significant efforts have been made to study the efficacy of eHealth programs in cohort studies, there is a dearth of literature on the demographics and attitude-related disparity between members of the general population who have or do not have diabetes. In addition, many of the research studies gathered data from people who had already used specific services to assess the extent of user experience. The expectations and attitudes of those who had never used the technology were not addressed. These perspectives are imperative in the coming era, where the growing demand for health care services will prompt organizations to leverage eHealth for cost-effectiveness and efficient delivery of care. To date, no studies have been conducted at the population level to understand the readiness, acceptability, and attitudes of the general public toward eHealth services for diabetes care. eHealth was one of the most sought-after technologies in the COVID-19 pandemic phase, where many countries tried to deliver diabetes care through eHealth [20]. However, the patients were unwilling to accept the platform even in countries that had an integrated eHealth framework. The most accepted eHealth service is receiving prescriptions for insulin and other medications [20]. Thus, these data clearly show that understanding the readiness and acceptance of eHealth is critical in the future to manage health care services during pandemics.

### Objectives

The objective of this study is to understand the (1) readiness and acceptance of eHealth services among the general population (with and without diabetes) at a national level, (2) perceived advantages and disadvantages of eHealth services, and (3) sociodemographic and clinical factors associated with eHealth readiness and acceptance. This will help organizations to consider various factors that stem from the patient’s needs or concerns when implementing eHealth services to avoid technology failure.

## Methods

### Participants

The detailed methodology of this study has been reported previously [21]. This study was part of a nationwide cross-sectional survey that was intended to understand the knowledge, attitude, and practices of the general public toward diabetes. Participants from the general public were randomly selected from a population registry database of Singapore comprising permanent residents and citizens through disproportionate random sampling (ethnicity and age). Surveys were conducted face-to-face by trained interviewers.

### Eligibility

Participants who were aged ≥18 years; who were citizens or permanent residents of Singapore; and who could understand English, Chinese, Malay, or Tamil were recruited. Those who were nonresidents or noncitizens, incapable of doing an interview, living outside the country, or institutionalized during the entire survey period were excluded from the study.

### Sample Size

Sample size calculation has been reported previously [21]. Briefly, the power calculations were based on the prevalence rates of knowledge in the general public and included calculations for binary proportions to determine overall sample sizes as well as those for subgroups to produce a margin of error of ≤0.05. A statistical power of 0.8 was targeted with the type 1 error limited to α of .05. The sample size was adjusted to accommodate deviations owing to random sampling. The study used 16 strata: four for ethnicity (Chinese, Malay, Indian, and others) and four for age groups (18-34 years, 35-49 years, 50-64 years, and ≥65 years). The sample was drawn in six replicates (total 5698) released at different intervals starting in February 2019. A target sample of 3000 was estimated to be sufficient to understand the knowledge, attitude, and practices of the general public. The study closed recruitment in September 2020 with a final response rate (total completed interviews / [total number of sample – eligible cases]) of 66.2%. The eligibility rate was 76.8%. The reasons for ineligibility included death, institutionalization (eg, incarceration), residing outside the country during the survey period, uncontactable, severe physical or mental condition that interfered with participation, and language barriers.

### Procedure

An invitation letter was sent to all participants 1 to 2 weeks before the intended home visit by a trained interviewer. The trained interviewer approached the households and captured the responses via computer-assisted personal interviews in a language preferred by the participant (English, Chinese, Malay, or Tamil). Regular quality checks were conducted on the data collected [21]. Written consent was obtained from all the participants before the survey. All study procedures were conducted in accordance with the ethical guidelines (Domain Specific Review Board reference 2018/00430). The study was suspended during the *circuit breaker* (heightened safe distancing measures that were implemented to prevent the spread of the virus. This included closure of schools, workplaces, other venues, and avoidance of interaction with those who do not live together) period in response to the pandemic (March 2020 to July 2020) and restarted in August 2020 with interviewers and respondents adhering to safe distancing measures and mask policy.

### Questionnaire

#### Sociodemographic Questionnaire

The information collected included age (as of last birthday), gender, ethnicity, employment status, educational qualification, average monthly personal income (including all allowances over the past 12 months), average household income, height, weight, BMI, and marital status.

#### Diabetes Questionnaire

The participants were asked two questions to ascertain their diabetes status: (1) “Have you ever been told by a doctor that you have Diabetes?” The response options were “yes,” “no,” or “I don’t know.” Those who answered “yes” were further probed with question (2) “What type of diabetes do you have?” The response options were “type 1 diabetes,” “type 2 diabetes,” “gestational diabetes,” “others,” and “I don’t know.” This analysis of the diabetes group included those who endorsed type 1 or type 2 diabetes.

#### eHealth Questionnaire

The eHealth questionnaire was administered after introducing the concept as “eHealth services refers to health care services (eg, Health Hub) delivered through internet which includes programmes such as online appointment booking, online prescription, online consultation with nurse/doctor/therapist, internet-based support programmes, online referrals, etc.”

##### Readiness

The readiness of the participants toward eHealth was assessed using a single statement, “I am not ready for eHealth.” Participants were asked to indicate the extent to which they agreed or disagreed with this statement. The responses were captured on a 5-point scale from “strongly agree” to “strongly disagree” with an option for “neutral” in the middle. For the regression analysis, the responses were grouped as “not ready” (ie, strongly agree or agree) versus “ready” (ie, neutral, strongly disagree, or disagree).

##### Acceptability

Acceptability of eHealth was assessed using two items: (1) “If you seek treatment for diabetes would you seek treatment delivered via the internet?” The responses were captured as five different options ranging from “definitely would” and “possibly would” to “definitely wouldn’t,” “definitely wouldn’t,” and “not sure.” For the logistic regression analysis, “definitely/possibly would” was combined into one category, “definitely/possibly wouldn’t” was subsumed into another group, and those who indicated “not sure” (n=95) were removed from the analyses. (2) The acceptance of specific health services was assessed using a stem question, which asked, “Overall, which type of service would you prefer to use if you experience diabetes-related health problems?” The services listed included consultation, receiving prescriptions, booking appointments, receiving health information, and referrals to other clinicians. The participants were asked to indicate their preferred methods to each listed service with one of the three response options “face-to-face,” “eHealth,” or “both eHealth and face-to-face*.*”

##### Perceived Advantages and Disadvantages of eHealth

This construct was captured using a series of statements with response options ranging from “strongly agree” to “strongly disagree” with an option for “neutral” in the middle (5-point scale). The items included “internet-based treatments” (1) are more convenient (do not have to travel to the clinic and can access anywhere), (2) save time, (3) are cost-saving, (4) ensure privacy and anonymity (personal information is kept confidential), (5) save from embarrassment related to face-to-face consultation, (6) might not be helpful for my health condition, (7) do not build clinician-patient rapport of face-to-face session, (8) require users to be computer literate, and (9) might not be credible. For bivariate chi-square analyses, the responses were grouped into three groups: strongly agree or agree, neutral, and strongly disagree or disagree.

#### Chronic Conditions Checklist

The chronic conditions checklist [22] captured 18 different chronic conditions (physical illnesses such as asthma, diabetes, stroke or major paralysis, high blood pressure, hyperlipidemia, arthritis or rheumatism, cancer, neurological conditions such as epilepsy or convulsions, Parkinson disease, congestive heart failure, heart diseases, back problems including disk or spine, stomach ulcer, chronic inflamed bowel, enteritis or colitis, thyroid diseases, kidney failure, migraine headaches, and chronic lung diseases [chronic bronchitis and emphysema]). The participants were asked to respond “yes” or “no” if they have ever been diagnosed with the condition. Diabetes, although captured in the list, was excluded when the number of chronic conditions was tabulated. The total number of chronic conditions was then grouped into *no chronic diseases*, *one chronic disease*, and *two or more chronic diseases*. The duration of each disease could be calculated through an additional question that asks, “How old were you when you were diagnosed?” However, only the duration of diabetes was used in this study. The quartile values were used for grouping the duration of diabetes into four categories (<4 years, 4-9 years, 10-18 years, and ≥19 years).

#### Analysis

To ensure the representativeness of the data to the general population, the following weights were used in the analysis: design weights to account for oversampling, poststratification weights for adjusting age and ethnicity distributions, and nonresponse weights. All statistical analyses were performed using Stata MP version 15 (StataCorp LLC), and all descriptive, chi-square, and regression analyses were weighted using survey weights to account for the complex survey design. Descriptive analysis was performed for the variables, and the data were represented as the frequency and weighted percentage of the events. First, bivariate chi-square analyses were performed to compare the sociodemographic characteristics of the diabetes and nondiabetes groups. Second, bivariate chi-square analyses were also conducted to examine how the diabetes and nondiabetes groups differed from each other in terms of the perceived advantages or disadvantages of eHealth. Third, within the full sample, multivariable logistic regression analyses (multiple predictor variables and a single binary outcome variable) were then conducted to determine significant sociodemographic factors (ie, age, gender, ethnicity, education, marital status, monthly personal income, employment, BMI, diabetes diagnosis, and chronic physical conditions) associated with readiness for eHealth (ready vs not ready) and acceptance of eHealth (would not vs would accept). The estimated odds ratios (ORs) for each predictor variable of the multivariable regression models were adjusted for other observable variables or potential confounders entered within the model. Finally, to examine the sociodemographic variables associated with readiness or acceptance within individuals with diabetes, a series of bivariate chi-square or Mann-Whitney U analyses were conducted before the estimation of multivariable logistic regression models. Owing to the limited sample size of individuals who endorsed having diabetes, only variables that showed a significant association in the aforementioned bivariate chi-square analyses were included in the final multivariable logistic models. Missing, refused, or do not know responses were removed listwise, as is the default in multivariable logistic regression models and bivariate chi-square analyses.

## Results

### Sociodemographic Characteristics of the Sample

In total, 2895 participants were recruited from the general population (screened=5698; response rate 66.2%; eligibility rate 76.8%), of which 15.06% (436/2895) had diabetes, whereas the 84.94% (2459/2895) did not have diabetes. An approximately equal number of all age groups, gender, and ethnicity (Multimedia Appendix 1, Table S1) were recruited. Most participants were married (61.7%) and employed (70.5%). Of the participants, 47.6% had no other chronic illnesses (excluding diabetes), 27.3% had at least one chronic disease, and 24.9% had multiple comorbidities (two or more chronic diseases). Detailed information is presented in Multimedia Appendix 1, Table S1. The diabetes and nondiabetes groups differed significantly in most of the characteristics analyzed (*P*<.001).

### Readiness and Acceptance Toward eHealth

Nearly half of the participants acknowledged that they were not ready for eHealth (47.3%). Readiness varied significantly between the nondiabetes and diabetes samples, with the latter showing significantly lower readiness (Table 1; 54.9% vs 24%; *P*<.001). A lower acceptance level toward eHealth was observed overall (28%), with the majority (68.4%) unwilling to use eHealth for diabetes care. The diabetes group showed significantly lower acceptance (12.1%) than the nondiabetes group (29.6%; *P*<.001).

**Table 1:** Readiness toward eHealth and acceptability of eHealth services for diabetes care.

Response categories		Overall (N=2895)	Nondiabetes (n=2459)	Diabetes (n=436)	*P* value	
**Readiness toward eHealth technology (“I am not ready for eHealth”), n (%)^a^**						<.001
	Not ready	1460 (47.3)	1132 (44.4)	328 (75.7)		
	Ready	1419 (52.1)	1314 (54.9)	105 (24)		
	Do not know or refused^b^	16 (0.6)	13 (0.7)	3 (0.3)		
**Acceptance of eHealth for diabetes care (“If you seek treatment for diabetes, would you seek treatment delivered via internet?”), n (%)**						<.001
	Definitely or possibly would	692 (28)	642 (29.6)	50 (12.1)		
	Definitely or possibly wouldn't	2100 (68.4)	1733 (67.1)	367 (81.8)		
	Not sure^b^	95 (3.3)	78 (3)	17 (5.9)		
	Do not know or refused	8 (0.3)	6 (0.3)	2 (0.2)		

^a^Weighted percentage.

^b^Do not know or refused or not sure options were not included in the bivariate chi-square analysis.

### Acceptance Toward Specific eHealth Services

A strong preference for face-to-face sessions over eHealth was observed, especially among the diabetes group for most of the services studied. eHealth services were acceptable for the majority of the participants (overall sample, Table 2) for booking appointments (eHealth only: 33.3%; both eHealth or face-to-face: 34.1%) and for receiving health information (eHealth only: 23%; both: 39.8%). Although the nondiabetes group had roughly the same general acceptance level as the overall sample for eHealth, the diabetes group differed significantly in their acceptance rates, preferring face-to-face services (eg, 55.7% for booking appointments and 63.4% for receiving health information; *P*<.001). Acceptance was lower for other services in the overall sample, such as receiving prescriptions for medications (eHealth: 11.3%; both: 24.8%), consultation with clinicians (eHealth only: 1.3%; both: 13.9%), and receiving referral to other doctors (eHealth only: 13.2%; both: 33%). The diabetes group reported significantly lower acceptance rates for all of the previously mentioned services than the nondiabetes group (*P*<.001). A strong preference for face-to-face services was observed for consultation with clinicians, with 92.6% of the diabetes group preferring face-to-face consultation sessions, compared with 83.5% of the nondiabetes group (*P*<.001).

**Table 2:** Comparison of acceptance of various eHealth services between diabetes and nondiabetes groups.

Response categories			Overall		Nondiabetes		Diabetes		*P* value	
**Booking appointment, n (%)**										<.001
	Face-to-face only	1076 (32.1)		794 (29.7)		282 (55.7)				
	eHealth only	854 (33.3)		800 (35.6)		54 (10.8)				
	Both	953 (34.1)		858 (34.3)		95 (31.4)				
	Do not know, refused, or missing^a^	12 (0.5)		7 (0.3)		5 (2.1)				
**Getting prescriptions, n (%)**										<.001
	Face-to-face only	1957 (63.5)		1599 (61.9)		358 (79.3)				
	eHealth only	280 (11.3)		259 (11.7)		21 (6.4)				
	Both	648 (24.8)		595 (26.0)		53(13.1)				
	Do not know, refused, or missing	10 (0.4)		6 (0.3)		4 (1.2)				
**Consultation, n (%)**										<.001
	Face-to-face only	2521 (84.3)		2118 (83.5)		403 (92.6)				
	eHealth only	34 (1.3)		33 (1.5)		1 (0.1)				
	Both	329 (13.9)		301 (14.7)		28 (6.1)				
	Do not know, refused, or missing	11 (0.4)		7 (0.3)		4 (1.2)				
**Health information, n (%)**										<.001
	Face-to-face only	1262 (36.7)		955 (34)		307 (63.4)				
	eHealth only	588 (23)		555 (24.4)		33 (8.6)				
	Both	1033 (39.8)		942 (41.2)		91 (25.9)				
	Do not know, refused, or missing	12 (0.5)		7 (0.3)		5 (2.1)				
**Referrals to other doctors, n (%)**										<.001
	Face-to-face only	1704 (53.3)		1359 (51.4)		345 (72.2)				
	eHealth only	377 (13.2)		356 (14.2)		21 (3.6)				
	Both	801 (33.0)		735 (34)		66 (23)				
	Do not know, refused, or missing	13 (0.5)		9 (0.5)		4 (1.2)				

^a^Do not know or refused options and missing data were not included in the bivariate chi-square analysis.

### Perceived Advantages and Disadvantages of eHealth

**Figure 1:**
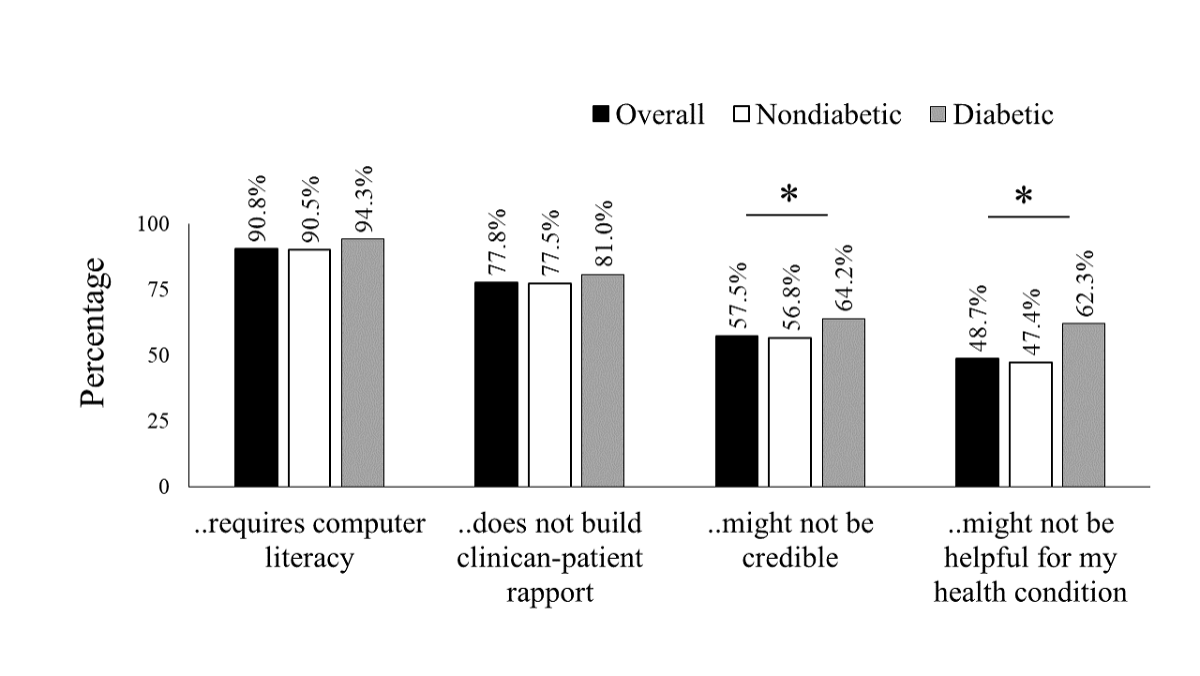
Disadvantages of eHealth reported by the participants. The asterisk represents statistically significant values (*P*<.05).

**Figure 2:**
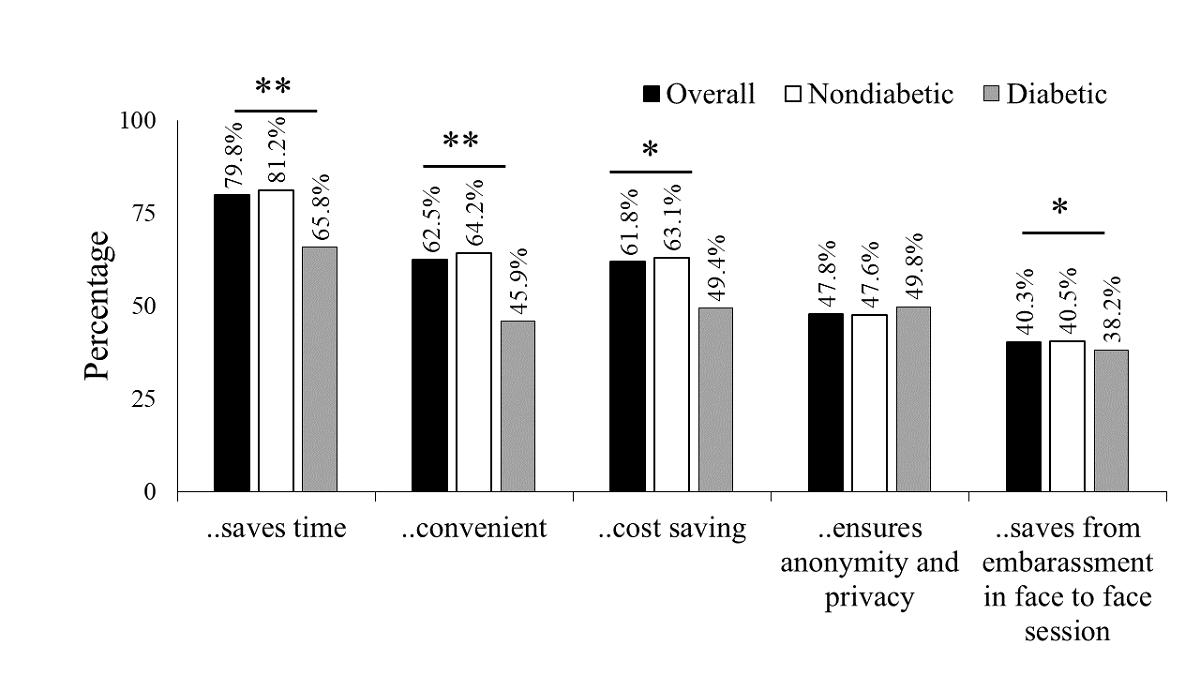
Advantages of eHealth as reported by the participants. Statistical significance is marked with asterisk (*P<.05; **P<.001).

The most common disadvantages cited were the requirement of computer literacy to use the eHealth service (90.8%; Figure 1), followed by the perception that eHealth did not build clinician-patient rapport in comparison with the face-to-face sessions (77.8%), lack of confidence about the credibility of the eHealth services (57.5%), and the appropriateness of the eHealth services for their specific medical condition (48.7%). A significantly higher proportion of the diabetes group perceived the credibility of the eHealth services (64.2%) and appropriateness of the app for their specific medical condition (62.3%) as disadvantages compared with the nondiabetes group (56.8% and 47.4%, respectively). As a whole, the advantages cited included (1) eHealth services save time (79.8%; Figure 2), (2) it was more convenient than face-to-face sessions (62.5%), and (3) it was cost-saving (61.8%). Overall, the diabetes group perceived significantly lower advantages than the nondiabetes group, except that eHealth ensured anonymity and privacy. Nearly half of the participants in the diabetes (49.8%) and nondiabetes groups (47.6%) reported that eHealth ensures privacy and anonymity. Participants who showed acceptance (endorsed definitely or possibly would use the service) perceived the advantages significantly higher than those with lower acceptance (*P*<.001; Multimedia Appendix 1, Table S2).

### Factors Associated With eHealth Readiness and Acceptance

#### Factors Associated With eHealth Readiness

Frequencies and weighted percentage counts between sociodemographic variables and readiness toward eHealth are presented in Multimedia Appendix 1, Table S3. The results of the logistic regression analyses are presented in Table 3. The results indicated that age, ethnicity, and education status were associated with eHealth readiness (Multimedia Appendix 1, Table S3 for bivariate analysis). Older adults (aged ≥35 years) had lower odds of endorsing readiness for eHealth than those aged 18-34 years (*P*<.001). With regard to ethnicity, Indians had lower odds of eHealth readiness (OR 0.71, 95% CI 0.54-0.94; *P*=.02) than individuals of Chinese ethnicity. Those with primary school education or below (OR 0.24, 95% CI 0.14-0.44; *P*<.001) and secondary school education (OR 0.48, 95% CI 0.31-0.75; *P*=.001) had lower odds of eHealth readiness than those who had completed a degree or above.

**Table 3:** Factors affecting eHealth readiness.

Sociodemographic factors			Odds ratio^a^ (95% CI)		*P* value
**Age group (years)**					
	18-34 (reference)	—^b^		—	
	35-49	0.29 (0.19-0.45)		<.001	
	50-64	0.14 (0.08-0.22)		<.001	
	≥65	0.05 (0.03-0.11)		<.001	
**Gender**					
	Female (reference)	—		—	
	Male	1.24 (0.93-1.66)		.14	
**Ethnicity**					
	Chinese (reference)	—		—	
	Malay	1.27 (0.95-1.69)		.10	
	Indian	0.71 (0.54-0.94)		.02	
	Others	1.40 (0.89-2.21)		.15	
**Education**					
	Primary and below	0.24 (0.14-0.44)		<.001	
	Secondary school	0.48 (0.31-0.75)		.001	
	Preuniversity or junior college	1.04 (0.54-2.02)		.90	
	Vocational institute or Institute of Technical Education	0.84 (0.47-1.50)		.56	
	Diploma	0.98 (0.64-1.49)		.92	
	Degree, professional certification, and above (reference)	—		—	
**Marital status**					
	Single	0.92 (0.60-1.39)		.68	
	Married or cohabiting (reference)	—		—	
	Divorced or separated	0.71 (0.34-1.47)		.35	
	Widowed	1.30 (0.55-3.07)		.55	
**Employment**					
	Employed (reference)	—		—	
	Economically inactive	0.85 (0.55-1.34)		.49	
	Unemployed	0.98 (0.48-2.01)		.95	
**Monthly income in SGD (US $)**					
	Below 2000 (1481; reference)	—		—	
	2000-3999 (1481-2960)	0.97 (0.66-1.42)		.86	
	4000-5999 (2961-4441)	1.07 (0.64-1.77)		.80	
	6000-9999 (4442-7402)	1.48 (0.80-2.74)		.21	
	≥10,000 (7403)	1.98 (0.97-4.04)		.06	
	No income	1.07 (0.57-2.01)		.82	
**BMI**					
	Underweight	1.06 (0.56-2.00)		.85	
	Normal range (reference)	—		—	
	Overweight	0.75 (0.55-1.04)		.08	
	Obese	1.16 (0.76-1.78)		.50	
**Diabetes diagnosis**					
	No diabetes (reference)	—		—	
	Has diabetes	0.73 (0.46-1.16)		.18	
**Number of chronic conditions (excluding diabetes)**					
	No chronic diseases (reference)	—		—	
	One chronic disease	0.80 (0.58-1.13)		.20	
	Two or more chronic diseases	1.11 (0.77-1.59)		.57	

^a^Odds ratio >1 indicates higher likelihoods of endorsing readiness for eHealth.

^b^Values not estimated for reference groups.

#### Factors Associated With eHealth Acceptance

Compared with younger adults (aged 18-34 years), adults in older age groups (aged ≥35 years) were less likely to accept eHealth in the overall sample (Table 4). Indians had lower odds of accepting eHealth (OR 0.54, 95% CI 0.41-0.72; *P*<.001) than Chinese participants. Those who were single (OR 0.62, 95% CI 0.42-0.91; *P*=.01) were less likely to accept eHealth than those who were married or cohabiting. Those with a preuniversity or junior college education (OR 1.90, 95% CI 1.03-3.51; *P*=.04) were more likely to accept eHealth than those with degrees and above. In contrast, those with primary and lower education levels (OR 0.39, 95% CI 0.21-0.75; *P*=.01) were less likely to accept eHealth. People with no income were more likely to accept eHealth than those with income below SGD 2000 (US $1481; OR 1.88, 95% CI 1.06-3.34; *P*=.03). Compared with those with normal BMI, those underweight were less likely to accept eHealth (OR 0.47, 95% CI 0.27-0.83; *P*=.01). A detailed table with results from the bivariate analysis can be found in Multimedia Appendix 1, Table S4.

**Table 4:** Multivariable logistic regression model examining factors associated with eHealth acceptance within the overall sample.

Sociodemographic variables		Odds ratio^a^ (95% CI)	*P* value
**Age group (years)**			
	18-34 (reference)	—‑^b^	—
	35-49	0.56 (0.38-0.85)	.01
	50-64	0.31 (0.19-0.51)	<.001
	≥65	0.10 (0.05-0.22)	<.001
**Gender**			
	Female (reference)	—	—
	Male	1.24 (0.93-1.65)	.15
**Ethnicity**			
	Chinese (reference)	—	—
	Malay	1.06 (0.79-1.42)	.70
	Indian	0.54 (0.41-0.72)	<.001
	Others	0.73 (0.47-1.14)	.17
**Education**			
	Primary and below	0.39 (0.21-0.75)	.01
	Secondary school	0.69 (0.43-1.09)	.11
	Preuniversity or junior college	1.90 (1.03-3.51)	.04
	Vocational institute or Institute of Technical Education	0.57 (0.31-1.05)	.07
	Diploma	1.11 (0.73-1.68)	.62
	Degree, professional certification, and above (reference)	—	—
**Marital status**			
	Single	0.62 (0.42-0.91)	.01
	Married or cohabiting (reference)	—	—
	Divorced or separated	0.66 (0.30-1.42)	.28
	Widowed	0.98 (0.33-2.93)	.97
**Employment**			
	Employed (reference)	—	—
	Economically inactive	0.79 (0.51-1.24)	.31
	Unemployed	1.10 (0.58-2.10)	.77
**Monthly income in SGD (US $)**			
	Below 2000 (1481; reference)	—	—
	2000-3999 (1481-2960)	0.87 (0.58-1.31)	.51
	4000-5999 (2961-4441)	0.73 (0.43-1.24)	.24
	6000-9999 (4442-7402)	0.57 (0.31-1.08)	.08
	≥10,000 (7403)	1.88 (0.92-3.86)	.08
	No income	1.88 (1.06-3.34)	.03
**BMI**			
	Underweight	0.47 (0.27-0.83)	.01
	Normal range (reference)	—	—
	Overweight	0.75 (0.54-1.04)	.09
	Obese	0.67 (0.41-1.07)	.10
**Diabetes diagnosis**			
	No diabetes (reference)	—	—
	Has diabetes	0.65 (0.36-1.18)	.16
**Number of chronic conditions (excluding diabetes)**			
	No chronic diseases (reference)	—	—
	One chronic disease	0.76 (0.55-1.06)	.11
	Two or more chronic diseases	1.30 (0.88-1.90)	.19

^a^Odds ratio >1 indicates higher likelihood of accepting eHealth.

^b^Values not estimated for reference groups.

### Factors Associated With eHealth Readiness and Acceptance in Diabetes Subgroup

The frequencies of diabetes and sociodemographic variables and bivariate chi-square analyses are included in Tables S5 and S6 of Multimedia Appendix 1. Only significant correlates were entered into the final multivariate logistic regression model. Age, education status, and duration of diabetes were significantly associated with eHealth readiness in the diabetes group (Table 5). Those aged 50-64 years (OR 0.08, 95% CI 0.03-0.19; *P*<.001) and ≥65 years (OR 0.06, 95% CI 0.02-0.17; *P*<.001) showed lower odds of eHealth readiness compared with those aged 23-49 years. Participants with a diploma had lower odds of eHealth readiness (OR 0.21, 95% CI 0.06-0.76; *P*=.02) than those with an education level of degree or above. Those with a longer duration of diabetes had higher odds of readiness (4-9 years: OR 2.94, 95% CI 1.07-8.05, *P*=.04; 10-18 years: OR 4.03, 95% CI 1.46-11.18, *P*=.007) than those with <4 years of disease duration.

**Table 5:** Multivariable logistic regression model examining factors associated with eHealth readiness within the diabetes sample.

Sociodemographic factors		Odds ratio^a^ (95% CI)	*P* value
**Age (years)**			
	23-49 (reference)	—^b^	—
	50-64	0.08 (0.03-0.19)	<.001
	≥65	0.06 (0.02-0.17)	<.001
**Ethnicity**			
	Chinese (reference)	—	—
	Malay	0.98 (0.38-2.51)	.96
	Indian	0.88 (0.39-1.98)	.76
	Others	1.64 (0.39-6.90)	.50
**Education**			
	Primary and below	0.28 (0.07-1.09)	.07
	Secondary	0.40 (0.10-1.60)	.19
	Preuniversity or junior college	0.62 (0.11-3.52)	.59
	Vocational institute or Institute of Technical Education	1.44 (0.31-6.62)	.64
	Diploma	0.21 (0.06-0.76)	.02
	Degree, professional qualification, and above (reference)	—	—
**Marital status**			
	Single	2.18 (0.74-6.37)	.16
	Married or cohabiting (reference)	—	—
	Separated or widowed or divorced	0.74 (0.18-3.01)	.68
**Employment**			
	Employed (reference)	—	—
	Economically inactive	0.51 (0.15-1.77)	.29
	Unemployed	0.57 (0.17-1.91)	.36
**Duration of diabetes (years)**			
	<4 (reference)	—	—
	4-9	2.94 (1.07-8.05)	.04
	10-18	4.03 (1.46-11.18)	.01
	≥19	2.45 (0.73-8.27)	.15

^a^Odds ratio >1 indicates higher likelihood of endorsing readiness for eHealth.

^b^Values not estimated for reference groups.

Age and income were associated with eHealth acceptance and rejection in the diabetes sample (Table 6). Those aged 50-64 years (OR 0.24, 95% CI 0.08-0.75; *P*=.02) had higher odds of rejecting eHealth than those aged 23-49 years. Compared with the participants with income below SGD 2000 (US $1481), those with incomes between SGD 4000 (US $2961) and SGD 3999 (US $2960) were more likely to accept eHealth (OR 7.17, 95% CI 1.61-31.95; *P*=.01). The detailed data from the bivariate analysis are included in Multimedia Appendix 1, Tables S6 and S7.

**Table 6:** Factors associated with eHealth acceptance in the diabetes sample.

Sociodemographic factors			Odds ratio^a^ (95% CI)		*P* value
**Age (years)**					
	23-49 (reference)	—^b^		—	
	50-64	0.24 (0.08-0.75)		.02	
	≥65	0.24 (0.05-1.25)		.09	
**Employment**					
	Employed (reference)	—		—	
	Economically inactive	1.00 (0.24-4.23)		.99	
	Unemployed	0.69 (0.10-4.81)		.70	
**Personal income in SGD (US $)**					
	Below 2000 (1481; reference)	1.25 (0.43-3.61)		.68	
	2000-3999 (1481-2960)	7.17 (1.61-31.95)		.01	
	4000-5999 (2961-4441)	0.35 (0.05-2.50)		.30	
	6000-9999 (4442-7402)	1.72 (0.30-10.02)		.54	
	≥10,000 (7403)	0.24 (0.04-1.41)		.11	
	No income	1.25 (0.43-3.61)		.68	

^a^Odds ratio >1 indicates higher likelihood of accepting eHealth.

^b^Values not estimated for reference groups.

## Discussion

### Principal Findings and Implications

This study sheds light on the readiness, acceptance, and attitudes of the population, with and without diabetes, toward eHealth services. Nearly half of the population was not ready for the technology, which was more evident in people with diabetes (75.7%). The acceptance level was very low (12% in people with diabetes vs 29.6% in those without diabetes). Age, ethnicity, education, marital status, income, and duration of disease were associated with readiness or acceptance.

Overall, the participants favored face-to-face sessions over eHealth for most of the services surveyed. A strong preference for face-to-face sessions for consultation with clinicians was observed in 84% of the participants and 92% of the diabetes sample. Similar results were noted in other studies in patients with diabetes [23] and in patients with mental illness [24]. It is possible that patients with diabetes feel that diabetes is a complex disease with chances for further health complications; thus, periodic face-to-face sessions with their care providers are required. These sessions answer their queries and help them make important care decisions through discussions with their clinicians. Such interactions and rapport are important in the adaptation to medical conditions and engagement in treatment [25]. In agreement with this, 62.3% of the diabetes group in this study felt that eHealth might not be suitable for their condition, and the majority of the participants in both groups (diabetes and nondiabetes) felt that eHealth did not build clinician-patient rapport to the same extent as face-to-face sessions. Previous studies have identified potential challenges in patient-clinician interaction within the eHealth milieu, wherein 88% of the participants felt that eHealth does not build the same rapport as face-to-face sessions and sharing doctor’s phone number or personal email could improve the clinician-patient rapport in the eHealth framework [26]. However, only 2% of the doctors were willing to share their phone number or email address [27] with the patients. Thus, in the absence of additional measures, individuals who value clinician-patient rapport are unlikely to accept eHealth as a replacement for face-to-face sessions [23].

A lower perception of ease of use was also observed in the sample. The most cited disadvantage was the requirement of computer skills, which was identified as a factor that affects the behavioral outcome (perceived ease of use and intention to use the technology) in the Technology Acceptance Model [16]. This observation has also been highlighted in previous studies [24,28]. The usage and navigation of the various functions of apps require training and support, which can adversely affect user acceptance, especially in older adults. This can be addressed by constant training and support by health care staff [29]. Perceived benefits are another factor that determines the acceptance of eHealth services [16,28], especially among people with diabetes [29]. Although the majority of the nondiabetes group agreed that eHealth was convenient and saves time and cost, the diabetes sample showed a significantly lower perception of advantages that could add to their decision to reject the services. Apart from these factors, a user’s positive experience with eHealth services and eHealth literacy are important factors that can improve acceptance rates [29]. Thus far, none of the diabetes-related services in Singapore have been offered through eHealth platforms, thereby leaving the participants with no previous experience or knowledge of the technology. This could be the reason for the lower perception of benefits.

Age, education status, income, ethnicity, disease duration, BMI, and marital status were identified as factors associated with readiness and acceptance. We observed that a longer duration of diabetes was associated with higher odds of readiness but not acceptance in patients with diabetes, confirming previous reports. It is postulated that patients with a longer duration of diabetes prefer more personalized care, which involves regular face-to-face discussions with their health care team. In general, they lack trust in new technologies and are reluctant to embrace the new technology that has a lot of uncertainty to replace the comfort of face-to-face sessions [23]. However, as they tend to show readiness toward eHealth, the technology could be introduced slowly alongside their face-to-face session to give a positive experience with the technology that will influence their attitude toward eHealth. Jiang et al [30] studied the sociodemographic factors associated with the acceptance of eHealth in the management of chronic diseases (cancer), where the patients used eHealth for self-management. The study identified similar sociodemographic factors as determinants of eHealth acceptance. Younger adults and those with higher education exhibited higher eHealth literacy and thus had better chances of acceptance and outcome from eHealth use [31]. Gordon et al [32] and Eszes et al [33] also observed social determinants, such as age and ethnicity, to affect eHealth usage and acceptance. Thus, such social determinants should be given attention to avoid health care disparities in underprivileged groups. Overall, the present population in this study had an unfavorable attitude toward eHealth for diabetes care, and this was stronger among patients with diabetes. This is different from the reports from cohort studies where moderate or high levels of acceptance of specific eHealth apps were noted [24,34]. However, all these studies captured acceptance after implementing a specific eHealth program under the care of the attending health care professional, where the patients experienced remote glucose monitoring, diet, and/or physical activity monitoring. It is unclear whether these sessions supplemented their routine face-to-face sessions or replaced them. The participants in this study were technology naïve, as most of the services they encountered were offered face-to-face in the diabetes clinics with no remote monitoring in place. Hence, the lack of previous experience and literacy in eHealth might affect their perceptions and attitudes toward technology.

Overall, the diabetes sample showed a significantly lower readiness and acceptance of eHealth compared with the nondiabetes group. This can be explained based on the differences in sociodemographic characteristics between the two groups (Multimedia Appendix 1, Table S1). Most of the diabetes sample was older adults (aged >34 years), and only 1.8% of the participants were in the 21-34 years age group compared with 32.7% in the nondiabetes group. Nearly half of the diabetes sample (49.1% vs 24.5%) were aged between 50 and 64 years. Our results showed that adults who were aged ≥34 years had higher odds of not favoring eHealth, and the odds increased with age. Thus, the sociodemographic profile of the diabetes sample matches the archetype of individuals who tend not to favor eHealth. The diabetes sample also had a higher proportion of participants from the Indian ethnicity (15.1% vs 7.9%) and those reporting no income (10.9% vs 6.6%), all of which were associated with lower acceptance or readiness toward eHealth. It is also possible that those with diabetes had built a stronger therapeutic relationship with the clinical care team and thus were unwilling to accept new technology, as they feared that it would disrupt their relationship with the clinical team [23]. An in-depth qualitative study that captures the barriers to and facilitators of eHealth acceptance in this group is desirable to understand the needs of this group before introducing eHealth into their routine care.

It is possible that some participants were unaware of existing eHealth platforms in Singapore when answering the questions, and the survey did not capture this information, which is a limitation of this study. The questionnaire used in the study was developed by the study team and has not been validated previously. The diagnosis and duration of the disease were captured through a self-reported measure, which is a limitation of this study. For the duration of diabetes, the participants reported the age at which they were first diagnosed with diabetes, which was subjected to recall bias. It is currently unclear why a significantly higher proportion of patients with diabetes were not in favor of this technology. Age could play an important role, as older participants tend to reject eHealth. With the aging population in Singapore, it is essential to understand the barriers to and facilitators of eHealth acceptance to avoid the underutilization of services by this group. Future research should focus on an in-depth qualitative analysis of the population with and without diabetes with specific age groups to understand the reasons for the lack of acceptance of the technology.

It is also possible that people’s perceptions and attitudes toward eHealth evolved during the pandemic period, as many services were disrupted as a consequence of the COVID-19 pandemic. We recruited only a small proportion of the participants (n=16) during this period, and therefore, it was unlikely to have an impact on the results. Globally, patients with diabetes have multiple complications and hyperglycemic episodes with no or limited access to medications or other health care platforms [20]. Although eHealth is a viable option, it is not available to the majority of people worldwide. On the basis of these experiences, the international consensus calls for a transformation of diabetes care to steer toward eHealth rather than going back to the pre–COVID-19 era to prepare better for future disasters [20].

Lower eHealth readiness and acceptance can be a significant barrier to the digitalization of health care services. The general public’s eHealth literacy needs to be improved through education and communication before implementing any eHealth services to avoid patient and clinician distress. As patients value clinician-patient rapport, an organizational-level effort is required to improve the clinician-patient rapport in the eHealth framework and to assure patients that comparable care would be delivered under both platforms. Thus, a carefully planned deployment of eHealth to supplement face-to-face sessions rather than replace them would be ideal for improving acceptance and reducing patient dissatisfaction. A gradual transition based on an individual’s preferences, capabilities, and needs would result in people feeling comfortable with the technology.

### Conclusions

This study showed a negative attitude toward eHealth, with the majority unwilling to use eHealth for their diabetes care. The highest acceptance was noted for booking appointments on the web and receiving health information, whereas face-to-face sessions were preferred for the rest of the services. Participants were aware that eHealth saves time and is convenient; nonetheless, the lower perception of the benefits in patients with diabetes and higher perception of disadvantages is a challenge in accepting the technology for diabetes care. A strong preference for face-to-face sessions was observed with a larger proportion of participants with diabetes, citing the reason that eHealth might not be suitable for their health condition. The lack of clinician-patient rapport, requirement of computer skills, and privacy were highlighted by most of the participants, which needs to be addressed through awareness programs to improve acceptance. Younger age, higher education, marital status, BMI, higher income, and ethnicity were associated with eHealth readiness or acceptance. Attention must be given to the socioeconomic group who are unlikely to use the technology so that they are not affected by health and health care disparities.

## Appendix

Multimedia Appendix 1 Supplementary information with detailed analysis.

## Abbreviations

OR: odds ratio
