# Supplementary material for: Readiness and Acceptance of eHealth Services for Diabetes Care in the General Population: Cross-sectional Study
Source: J Med Internet Res. 2021 Sep 2;23(9):e26881. doi: 10.2196/26881 (PMC8446836; doi:10.2196/26881)
Supplement: Multimedia Appendix 1 [file jmir_v23i9e26881_app1.docx]

**Supplementary Section**

**Table S1:** Sociodemographic characteristics of the sample

| **Socio demographic information** | **Overall**  **n=2895** | | **Non-diabetic**  **n=2459** | | **Diabetic**  **n=436** | | ***P* values** |
| --- | --- | --- | --- | --- | --- | --- | --- |
|  | n | %^a^ | n | % | n | % |  |
|  |  |  |  |  |  |  |  |
| **Age group**  18-34 | 823 | 29.9% | 817 | 32.7% | 6 | 1.8% | **< .001** |
| 35-49 | 719 | 28.2% | 670 | 29.8% | 49 | 12.3% |  |
| 50-64 | 774 | 26.8% | 591 | 24.5% | 183 | 49.1% |  |
| 60 and above | 579 | 15.1% | 381 | 13.0% | 198 | 36.9% |  |
| **Gender**  Female | 1,474 | 51.6% | 1258 | 52.23% | 216 | 44.9% | **.09** |
| Male | 1,421 | 48.5% | 1201 | 47.77% | 220 | 55.2% |  |
| **Ethnicity**  Chinese | 796 | 75.8% | 731 | 76.9% | 65 | 64.9% | **< .001** |
| Malay | 974 | 12.7% | 811 | 12.1% | 163 | 18.7% |  |
| Indian | 918 | 8.6% | 725 | 7.9% | 193 | 15.1% |  |
| Others | 207 | 2.9% | 192 | 3.0% | 15 | 1.4% |  |
| **Education**  Primary and Below | 637 | 20.4% | 456 | 18.3% | 181 | 40.8% | **< .001** |
| Secondary School | 684 | 20.3% | 552 | 19.7% | 132 | 26.6% |  |
| Pre-U/Junior College | 126 | 4.8% | 112 | 5.0% | 14 | 2.3% |  |
| Vocational Institute/ITE | 267 | 6.6% | 241 | 6.8% | 26 | 5.2% |  |
| Diploma | 479 | 18.5% | 442 | 19.0% | 37 | 12.8% |  |
| Degree, professional certification, and above | 702 | 29.5% | 656 | 31.2% | 46 | 12.5% |  |
| **Marital status**  Single | 731 | 29.2% | 704 | 31.7% | 27 | 4.7% | **< .001** |
| Married/cohabiting | 1,860 | 61.7% | 1,531 | 60.0% | 329 | 78.1% |  |
| Divorced/separated | 154 | 5.0% | 131 | 5.0% | 23 | 4.9% |  |
| Widowed | 149 | 4.1% | 92 | 3.3% | 57 | 12.3% |  |
| **Employment**  Employed | 1,933 | 70.5% | 1,731 | 72.4% | 202 | 51.1% | **<.001** |
| Economically inactive | 829 | 25.4% | 617 | 23.7% | 212 | 41.8% |  |
| Unemployed | 133 | 4.1% | 111 | 3.8% | 22 | 7.1% |  |
| **Monthly Income (SGD)**  Below 2,000 | 1,236 | 38.3% | 982 | 36.8% | 254 | 54.1% | **.001** |
| 2,000 to 3,999 | 698 | 23.9% | 627 | 24.6% | 71 | 17.3% |  |
| 4,000 to 5,999 | 318 | 12.8% | 295 | 13.2% | 23 | 8.3% |  |
| 6,000 to 9,999 | 183 | 7.8% | 167 | 8.2% | 16 | 4.0% |  |
| 10,000 & above | 117 | 5.7% | 104 | 5.9% | 13 | 3.3% |  |
| No income | 219 | 7.0% | 173 | 6.6% | 46 | 10.9% |  |
| ^b^Don’t Know/Refused | 124 | 4.5% | 111 | 4.7% | 13 | 2.1% |  |
| **Body Mass Index**  Underweight | 151 | 6.9% | 146 | 7.4% | 5 | 2.3% | **< .001** |
| Normal Range | 1,263 | 53.4% | 1,148 | 55.6% | 115 | 31.4% |  |
| Overweight | 858 | 26.6% | 702 | 25.3% | 156 | 38.8% |  |
| Obese | 420 | 9.0% | 325 | 8.1% | 95 | 17.4% |  |
| ^b^Don’t Know/Refused | 203 | 4.1% | 138 | 3.5% | 65 | 10.1% |  |
| **Number of chronic conditions (excluding diabetes)** | | | | | | | |
| No chronic diseases | 1,324 | 47.6% | 1,243 | 50.9% | 81 | 47.6% | **< .001** |
| One chronic disease | 780 | 27.3% | 679 | 27.4% | 101 | 27.3% |  |
| Two or more chronic diseases | 783 | 24.9% | 531 | 21.4% | 252 | 24.9% |  |
| ^b^Missing | 8 | 0.3% | 6 | 0.3% | 2 | 0.3% |  |

^a^ the % represented weighted analysis; ^b^ Don’t Know/Refused options and missing data were not included in bivariate chi-square analyses.

**Table S2:** Perceived advantages and disadvantages of e-health by acceptance

| **Sociodemographic Factors** | **^a^Acceptance (Yes)** | | **Acceptance (No)** | | ***P* value** |
| --- | --- | --- | --- | --- | --- |
|  | n | % | n | % |  |
|  |  |  |  |  |  |
| **Internet based treatments are more convenient ( do not have to travel to clinic, can access anywhere)** | | | | |  |
| Strongly agree/agree | 597 | 87.1% | 1,101 | 52.6% | **< .001** |
| Neutral | 67 | 8.8% | 365 | 18.3% |  |
| Strongly disagree/disagree | 28 | 4.1% | 547 | 27.2% |  |
| ^b^ Missing/Don’t Know/Refused | 0 | 0.0% | 85 | 1.8% |  |
| **Internet based treatments save time** | | | | |  |
| Strongly agree/agree | 655 | 93.9% | 1,516 | 73.8% | **< .001** |
| Neutral | 27 | 4.5% | 229 | 11.1% |  |
| Strongly disagree/disagree | 10 | 1.5% | 267 | 13.5% |  |
| ^b^ Missing/Don’t Know/Refused | 0 | 0 | 88 | 1.6% |  |
| **Internet based treatments are cost saving** | | | | |  |
| Strongly agree/agree | 571 | 78.4% | 1,188 | 55.2% | **< .001** |
| Neutral | 93 | 17.7% | 389 | 22.3% |  |
| Strongly disagree/disagree | 22 | 3.3% | 313 | 16.3% |  |
| ^b^ Missing/Don’t Know/Refused | 6 | 0.6% | 210 | 6.3% |  |
| **Internet based treatments ensure privacy and anonymity (personal information is kept confidential)** | | | | |  |
| Strongly agree/agree | 432 | 55.9% | 1,026 | 44.5% | **< .001** |
| Neutral | 174 | 28.0% | 420 | 23.3% |  |
| Strongly disagree/disagree | 85 | 16.1% | 509 | 29.3% |  |
| ^b^ Missing/Don’t Know/Refused | 1 | 0.1% | 145 | 3.0% |  |
| **Internet based treatments saves from embarrassment related to face to face consultation** | | | | |  |
| Strongly agree/agree | 360 | 48.5% | 899 | 37.5% | **< .001** |
| Neutral | 183 | 27.6% | 394 | 20.3% |  |
| Strongly disagree/disagree | 149 | 23.9% | 767 | 41.0% |  |
| ^b^ Missing/Don’t Know/Refused | 0 | 0 | 40 | 1.1% |  |
| **Internet based treatments might not be helpful for my health condition** | | | | |  |
| Strongly agree/agree | 193 | 24.6 | 1,317 | 59.7 | **< .001** |
| Neutral | 246 | 36.3 | 389 | 22.8 |  |
| Strongly disagree/disagree | 248 | 38.0 | 315 | 15.6 |  |
| ^b^ Missing/Don’t Know/Refused | 5 | 1.2 | 79 | 1.9 |  |
| **Internet based treatments doesn’t build clinician-patient rapport of face to face session** | | | | |  |
| Strongly agree/agree | 446 | 62.9 | 1,792 | 84.3 | **< .001** |
| Neutral | 149 | 23.2 | 172 | 8.9 |  |
| Strongly disagree/disagree | 96 | 13.9 | 109 | 5.8 |  |
| ^b^ Missing/Don’t Know/Refused | 1 | 0.1 | 27 | 1.0 |  |
| **Internet based treatments requires computer literacy of the user** | | | | |  |
| Strongly agree/agree | 624 | 90.4 | 1,916 | 91.4 | **.47** |
| Neutral | 45 | 6.3 | 97 | 4.7 |  |
| Strongly disagree/disagree | 20 | 2.7 | 70 | 3.1 |  |
| ^b^ Missing/Don’t Know/Refused | 3 | 0.7 | 17 | 0.8 |  |
| **Internet based treatments might not be credible** | | | | |  |
| Strongly agree/agree | 282 | 39.6 | 1,327 | 65.0 | **< .001** |
| Neutral | 202 | 31.4 | 411 | 21.3 |  |
| Strongly disagree/disagree | 203 | 28.5 | 231 | 10.9 |  |
| ^b^ Missing/Don’t Know/Refused | 5 | 0.5 | 131 | 2.9 |  |

^a^Acceptance is scored” yes” if participant endorsed that they will definitely or possibly use the service; It is scored as no if the participant endorsed “definitely wouldn’t or possibly wouldn’t” use the service. Those who endorsed “Not Sure” (n=95) were not included in analyses.

^b^ Don’t Know/Refused options were not included in bivariate chi-square analyses.

**Table S3:** E-health readiness: frequency count and bivariate chi-square analyses

| **Sociodemographic factors** | **Not Ready**  **(Strongly Agree/Agree)** | | **Ready**  **(Neutral/Strongly Disagree/Disagree)** | | ***P* value** |
| --- | --- | --- | --- | --- | --- |
|  | n | % | n | % |  |
|  |  |  |  |  |  |
| **Age group** |  |  |  |  |  |
| 18-34 | 178 | 10.8% | 644 | 47.6% | **< .001** |
| 35-49 | 279 | 23.0% | 439 | 33.1% |  |
| 50-64 | 517 | 38.6% | 251 | 15.9% |  |
| 65 and above | 486 | 27.6% | 85 | 3.5% |  |
| **Gender** |  |  |  |  |  |
| Female | 802 | 54.2% | 662 | 48.9% | **.16** |
| Male | 658 | 45.8% | 757 | 51.1% |  |
| **Ethnicity** |  |  |  |  |  |
| Chinese | 395 | 77.4% | 395 | 74.3% | **< .001** |
| Malay | 492 | 11.8% | 476 | 13.6% |  |
| Indian | 497 | 9.0% | 417 | 8.3% |  |
| Others | 76 | 1.9% | 131 | 3.8% |  |
| **Education** |  |  |  |  |  |
| Primary and Below | 529 | 36.2% | 99 | 5.5% | **< .001** |
| Secondary School | 405 | 26.1% | 275 | 15.0% |  |
| Pre-U/Junior College | 49 | 3.6% | 76 | 5.9% |  |
| Vocational Institute/ITE | 94 | 4.4% | 172 | 8.6% |  |
| Diploma | 144 | 12.3% | 335 | 24.3% |  |
| Degree, professional certification, and above | 239 | 17.5% | 462 | 40.5% |  |
| **Marital status** |  |  |  |  |  |
| Single | 199 | 15.4% | 529 | 41.8% | **< .001** |
| Married/cohabiting | 1,033 | 71.1% | 817 | 53.4% |  |
| Divorced/separated | 102 | 6.8% | 51 | 3.2% |  |
| Widowed | 125 | 6.7% | 22 | 1.6% |  |
| **Employment** |  |  |  |  |  |
| Employed | 852 | 62.7% | 1,073 | 77.6% | **< .001** |
| Economically inactive | 541 | 33.2% | 280 | 18.2% |  |
| Unemployed | 67 | 4.1% | 66 | 4.2% |  |
| **Monthly Income (SGD)** | |  |  |  |  |
| Below 2,000 | 770 | 49.1% | 454 | 28.4% | **< .001** |
| 2,000 to 3,999 | 306 | 22.0% | 391 | 25.8% |  |
| 4,000 to 5,999 | 119 | 9.9% | 199 | 15.6% |  |
| 6,000 to 9,999 | 60 | 5.1% | 122 | 10.2% |  |
| 10,000 & above | 32 | 3.4% | 85 | 7.9% |  |
| No income | 127 | 7.6% | 90 | 6.3% |  |
| ^a^ Don’t Know/Refused | 46 | 2.9% | 78 | 6.0% |  |
| **Body Mass Index** |  |  |  |  |  |
| Underweight | 46 | 4.2% | 103 | 9.2% | **.001** |
| Normal Range | 579 | 50.5% | 678 | 56.1% |  |
| Overweight | 454 | 30.0% | 401 | 23.6% |  |
| Obese | 212 | 8.0% | 205 | 9.9% |  |
| Missing | 169 | 7.3% | 32 | 1.3% |  |
| **Diabetes Diagnosis** |  |  |  |  |  |
| No diabetes | 1,132 | 85.4% | 1,314 | 95.8% | **< .001** |
| Has diabetes | 328 | 14.6% | 105 | 4.2% |  |
| **Number of chronic conditions (excluding diabetes)** | | | |  |  |
| No chronic diseases | 548 | 38.5% | 773 | 56.0% | **< .001** |
| One chronic disease | 399 | 28.4% | 378 | 26.4% |  |
| Two or more chronic diseases | 513 | 33.1% | 268 | 17.7% |  |

^a^ Don’t Know/Refused options were not included in bivariate chi-square analyses.

**Table S4:** Acceptability of e-health: Frequency counts and bivariate chisquare analyses

| **Sociodemographic factors** | **Definitely/possibly would** | | **Definitely/possibly wouldn’t** | | **Not sure** | | ***P* value** |
| --- | --- | --- | --- | --- | --- | --- | --- |
|  | n | % | n | % | n | % |  |
|  |  |  |  |  |  |  |  |
| **Age group** |  |  |  |  |  |  | **< .001** |
| 18-34 | 302 | 43.9% | 492 | 24.2% | 28 | 32.2% |  |
| 35-49 | 213 | 33.6% | 481 | 26.3% | 25 | 24.0% |  |
| 50-64 | 136 | 18.4% | 602 | 29.5% | 32 | 37.3% |  |
| 65 and above | 41 | 4.1% | 525 | 20.0% | 10 | 6.5% |  |
| **Gender** |  |  |  |  |  |  | 0.44 |
| Female | 323 | 48.9% | 1,102 | 52.6% | 44 | 47.7% |  |
| Male | 369 | 51.1% | 998 | 47.4% | 51 | 52.4% |  |
| **Ethnicity** |  |  |  |  |  |  | .06 |
| Chinese | 223 | 77.9% | 544 | 74.9% | 25 | 74.7% |  |
| Malay | 230 | 12.1% | 680 | 12.5% | 36 | 15.0% |  |
| Indian | 173 | 6.5% | 713 | 9.4% | 29 | 8.7% |  |
| Others | 66 | 3.4% | 163 | 3.1% | 5 | 1.7% |  |
| **Education** |  |  |  |  |  |  | **< .001** |
| Primary and Below | 55 | 7.5% | 560 | 25.5% | 17 | 18.3% |  |
| Secondary School | 136 | 15.9% | 522 | 21.8% | 24 | 23.9% |  |
| Pre-U/Junior College | 44 | 7.9% | 80 | 3.7% | 2 | 0.6% |  |
| Vocational Institute/ITE | 64 | 6.0% | 194 | 7.0% | 8 | 3.3% |  |
| Diploma | 155 | 24.4% | 302 | 16.0% | 22 | 21.2% |  |
| Degree, professional certification, and above | 238 | 38.3% | 442 | 25.9% | 22 | 32.8% |  |
| **Marital status** |  |  |  |  |  |  | **.002** |
| Single | 243 | 36.9% | 465 | 26.1% | 22 | 30.0% |  |
| Married/cohabiting | 415 | 58.1% | 1,378 | 63.4% | 62 | 58.9% |  |
| Divorced/separated | 22 | 3.2% | 123 | 5.5% | 8 | 8.1% |  |
| Widowed | 12 | 1.9% | 133 | 5.0% | 3 | 3.0% |  |
| **Employment** |  |  |  |  |  |  | **.02** |
| Employed | 506 | 74.5% | 1,360 | 68.8% | 63 | 71.8% |  |
| Economically inactive | 148 | 20.7% | 650 | 27.6% | 27 | 18.0% |  |
| Unemployed | 38 | 4.8% | 90 | 3.6% | 5 | 10.2% |  |
| **Monthly Income (SGD)** | |  |  |  |  |  | **< .001** |
| Below 2,000 | 227 | 30.5% | 964 | 41.7% | 38 | 33.0% |  |
| 2,000 to 3,999 | 185 | 25.8% | 488 | 22.9% | 24 | 28.7% |  |
| 4,000 to 5,999 | 100 | 14.2% | 202 | 11.9% | 16 | 18.8% |  |
| 6,000 to 9,999 | 51 | 7.7% | 128 | 8.2% | 4 | 1.6% |  |
| 10,000 & above | 61 | 10.4% | 52 | 3.7% | 4 | 6.8% |  |
| No income | 50 | 8.0% | 161 | 6.4% | 8 | 10.7% |  |
| ^a^ Don’t Know/Refused | 18 | 3.4% | 105 | 5.2% | 1 | 0.4% |  |
| **Body Mass Index** |  |  |  |  |  |  | .16 |
| Underweight | 39 | 6.1% | 103 | 7.1% | 8 | 8.7% |  |
| Normal Range | 354 | 60.8% | 866 | 50.4% | 41 | 54.4% |  |
| Overweight | 195 | 23.4% | 637 | 28.0% | 24 | 24.1% |  |
| Obese | 83 | 7.8% | 318 | 9.5% | 17 | 8.9% |  |
| Missing | 21 | 1.9% | 176 | 5.0% | 5 | 3.9% |  |
| **Diabetes Diagnosis** | | |  |  |  |  | **< .001** |
| No diabetes | 642 | 96.1% | 1,733 | 89.1% | 78 | 83.5% |  |
| Has diabetes | 50 | 3.9% | 367 | 10.9% | 17 | 16.5% |  |
| **Number of chronic conditions (excluding diabetes)** | | | | | |  | **.03** |
| No chronic disease | 370 | 54.7% | 912 | 44.8% | 42 | 48.0% |  |
| One chronic disease | 184 | 24.8% | 568 | 28.2% | 28 | 31.1% |  |
| Two or more chronic diseases | 138 | 20.5% | 620 | 27.0% | 25 | 21.0% |  |

^a^ Don’t Know/Refused options were not included in bivariate chi-square analyses.

**Table S5:** e-health readiness in the diabetes subgroup: frequency count and bivariate chi-square analysis

| **Sociodemographic factors** | **Strongly agree/Agree**  **N=328** | | **Neutral/Strongly Disagree/Disagree**  **N=105** | | ***P* value** |
| --- | --- | --- | --- | --- | --- |
|  | n | % | n | % |  |
|  |  |  |  |  |  |
| **Age group** |  |  |  |  | **< .001** |
| ^a^ 23-49 | 19 | 3.5% | 36 | 47.5% |  |
| 50-64 | 137 | 53.0% | 44 | 36.3% |  |
| 65 and above | 172 | 43.5% | 25 | 16.2% |  |
| **Gender** |  |  |  |  | **.43** |
| Female | 177 | 48.6% | 38 | 33.3% |  |
| Male | 151 | 51.4% | 67 | 66.7% |  |
| **Ethnicity** |  |  |  |  | **.03** |
| Chinese | 52 | 67.6% | 13 | 57.1% |  |
| Malay | 126 | 18.2% | 36 | 19.9% |  |
| Indian | 140 | 13.4% | 51 | 19.9% |  |
| Others | 10 | 0.8% | 5 | 3.2% |  |
| **Education** |  |  |  |  | **< .001** |
| Primary and below | 162 | 48.4% | 11 | 11.1% |  |
| Secondary | 113 | 29.1% | 28 | 39.2% |  |
| Pre-U/Junior College |  |  |  |  |  |
| Vocational Institute/ITE |  |  |  |  |  |
| Diploma | 32 | 15.2% | 8 | 12.5% |  |
| Degree, professional certification, and above | 21 | 7.3% | 21 | 37.1% |  |
| **Marital status** |  |  |  |  | **.02** |
| Single | 16 | 3.3% | 11 | 9.1% |  |
| Married/cohabiting | 242 | 76.1% | 84 | 84.2% |  |
| Divorced/separated/Widowed | 70 | 20.5% | 10 | 6.7% |  |
| **Employment** |  |  |  |  | **<.0001** |
| Employed | 127 | 43.7% | 73 | 73.9% |  |
| Economically inactive | 184 | 48.9% | 27 | 19.6% |  |
| Unemployed | 17 | 7.4% | 5 | 6.5% |  |
| **Monthly Income (SGD)** |  |  |  |  | **.03** |
| Below 2,000 | 217 | 59.7% | 35 | 36.2% |  |
| 2,000 to 3,999 | 41 | 13.9% | 30 | 28.1% |  |
| 4,000 to 5,999 | 14 | 8.1% | 9 | 8.8% |  |
| 6,000 to 9,999 | 7 | 2.5% | 9 | 8.6% |  |
| 10,000 & above | 5 | 1.9% | 8 | 8.0% |  |
| No income | 38 | 12.2% | 7 | 6.8% |  |
| ^b^ Don’t Know/Refused | 6 | 1.7% | 7 | 3.5% |  |
| **Body Mass Index** |  |  |  |  | **.003** |
| Underweight | 4 | 1.6% | 1 | 4.8% |  |
| Normal Range | 81 | 31.8% | 34 | 30.5% |  |
| Overweight | 121 | 43.5% | 35 | 24.4% |  |
| Obese | 64 | 11.8% | 30 | 34.9% |  |
| Missing | 58 | 11.4% | 5 | 5.4% |  |
| **Number of chronic conditions (excluding diabetes)** | | | |  | **.29** |
| No chronic disease | 54 | 12.5% | 27 | 21.8% |  |
| One chronic disease | 78 | 25.9% | 23 | 24.1% |  |
| Two or more chronic diseases | 196 | 61.7% | 55 | 54.1% |  |
| **Duration of Diabetes** | |  |  |  |  |
| <4 years | 64 | 26.1% | 24 | 21.0% |  |
| 4-9 years | 76 | 22.3% | 34 | 37.2% | .09 |
| 10-18 years | 90 | 21.4% | 31 | 28.4% |  |
| >= 19 years | 98 | 30.2% | 16 | 13.5% |  |

^a^ All participants in the diabetes sample were 23 years and older

^b^ Don’t Know/Refused options were not included in bivariate chi-square analyses.

**Table S6**: Health acceptance in the diabetes sample: Frequency count and bivariate chi-square analyses

| **Sociodemographic factors** | **Definitely/Possibly Would (n=50)** | | **Definitely/Possibly Wouldn't (n=367)** | | ***P* value** |
| --- | --- | --- | --- | --- | --- |
|  | n | % | n | % |  |
|  |  |  |  |  |  |
| **Age group** |  |  |  |  | **.01** |
| 23-49* | 15 | 34.3% | 37 | 9.2% |  |
| 50-64 | 27 | 41.6% | 143 | 49.5% |  |
| 65 and above | 8 | 24.2% | 187 | 41.3% |  |
| **Gender** |  |  |  |  | .44 |
| Female | 22 | 37.5% | 188 | 47.2% |  |
| Male | 28 | 62.6% | 179 | 52.8% |  |
| **Ethnicity** |  |  |  |  | .68 |
| Chinese | 7 | 58.2% | 54 | 65.3% |  |
| Malay | 20 | 22.2% | 134 | 18.1% |  |
| Indian | 22 | 18.5% | 165 | 15.1% |  |
| Others | 1 | 1.0% | 14 | 1.6% |  |
| **Education** |  |  |  |  | .057 |
| Primary and below | 7 | 18.6% | 166 | 44.7% |  |
| Secondary, Vocational/ITE | 21 | 35.9% | 133 | 32.9% |  |
| Pre-U/Junior College, Diploma | 9 | 33.9% | 41 | 13.3% |  |
| Degree, professional certification, and above | 13 | 11.6% | 27 | 9.0% |  |
| **Marital status** |  |  |  |  | .92 |
| Single | 3 | 2.9% | 23 | 3.9% |  |
| Married/cohabiting | 43 | 78.3% | 269 | 78.0% |  |
| Divorced/separated/Widowed | 4 | 18.8% | 75 | 18.1% |  |
| **Employment** |  |  |  |  | **.035** |
| Employed | 34 | 72.9% | 158 | 46.6% |  |
| Economically inactive | 14 | 25.1% | 190 | 46.3% |  |
| Unemployed | 2 | 2.0% | 19 | 7.0% |  |
| **Monthly Income (SGD)** |  |  |  |  | **<.001** |
| Below 2,000 | 20 | 37.9% | 224 | 58.0% |  |
| 2,000 to 3,999 | 11 | 20.3% | 58 | 16.7% |  |
| 4,000 to 5,999 | 7 | 31.5% | 15 | 5.3% |  |
| 6,000 to 9,999 | 3 | 2.2% | 12 | 4.4% |  |
| 10,000 & above | 4 | 3.2% | 7 | 2.1% |  |
| No income | 2 | 1.6% | 42 | 11.6% |  |
| ^b^ Don’t Know/Refused | 3 | 3.3% | 9 | 1.9% |  |
| **Body Mass Index** |  |  |  |  | .35 |
| Underweight | 0 | 0.0% | 4 | 1.4% |  |
| Normal Range | 11 | 23.4% | 99 | 33.1% |  |
| Overweight | 19 | 35.0% | 129 | 38.8% |  |
| Obese | 17 | 32.7% | 76 | 16.1% |  |
| Missing | 3 | 9.0% | 59 | 10.5% |  |
| **Number of chronic conditions (excluding diabetes)** |  |  |  |  | .07 |
| No chronic disease | 12 | 18.4% | 65 | 13.3% |  |
| One chronic disease | 9 | 8.7% | 88 | 26.7% |  |
| Two or more chronic diseases | 29 | 72.9% | 214 | 60.0% |  |
| **Duration of Diabetes** | 12 | 12.5% | 70 | 25.1% | .52 |
| <4 years | 17 | 33.3% | 90 | 26.2% |  |
| 4-9 years | 10 | 22.0% | 107 | 23.1% |  |
| 10-18 years | 11 | 32.2% | 100 | 25.6% |  |
| ≥19 years | 12 | 12.5% | 70 | 25.1% |  |

^a^All participants in the diabetes sample were 23 years and older

^b^ Don’t Know/Refused options were not included in bivariate chi-square analyses.
